# Additive Effects of Levodopa and a Neurorestorative Diet in a Mouse Model of Parkinson’s Disease

**DOI:** 10.3389/fnagi.2018.00237

**Published:** 2018-08-03

**Authors:** Paula Perez-Pardo, Laus M. Broersen, Tessa Kliest, Nick van Wijk, Amos Attali, Johan Garssen, Aletta D. Kraneveld

**Affiliations:** ^1^Division of Pharmacology, Faculty of Science, Utrecht Institute for Pharmaceutical Sciences, Utrecht University, Utrecht, Netherlands; ^2^Nutricia Research, Utrecht, Netherlands

**Keywords:** Parkinson’s disease, levodopa, dietary intervention, motor-symptoms, non-motor symptoms

## Abstract

Though Parkinson’s disease (PD) clinical picture is generally dominated by motor impairment, non-motor symptoms, such as cognitive decline and gastrointestinal dysfunctions, may develop before motor symptoms and have major effects on quality of life. L-3,4-di-hydroxy-phenylalanine (Levodopa) is the most commonly used treatment of motor symptoms but has serious side-effects with prolonged use and does not stop the degenerative process. Moreover, gastrointestinal dysfunctions interfere with the absorption of levodopa and modify its effectiveness. Since most patients are on levodopa treatment, there is a need for combinational therapies that allow for an effective reduction of both motor and non-motor symptoms. We have recently shown that a diet containing precursors and cofactors required for membrane phospholipid synthesis, as well as prebiotic fibers, had therapeutic effects in a PD mouse model. We now investigate the effects of combined administration of the same diet together with levodopa in the rotenone model of PD. Mice were injected with rotenone or vehicle in the striatum. The dietary intervention started after full induction of motor symptoms. The effects of dietary intervention and oral treatment with different doses of levodopa were assessed weekly. Motor and cognitive functions were tested, intestinal transit was analyzed and histological examination of the brain and the colon was assessed. Our results confirm our previous findings that rotenone-induced motor and non-motor problems were alleviated by the Active diet (AD). Levodopa showed an additive beneficial effect on rotarod performance in rotenone-treated animals fed with the AD. No negative interaction effects were found between the AD and levodopa. Our findings suggest that the dietary intervention might confer additional clinical benefits on patients receiving levodopa treatment.

## Introduction

Parkinson’s disease (PD) is the second most frequent age-related neurodegenerative disorder for which there is no cure. It is hallmarked by the progressive degeneration of dopaminergic nigrostriatal neurons, with reductions in dopamine levels resulting in the characteristic motor impairment. Another characteristic of PD is the neuronal cytoplasmatic accumulation of alpha-synuclein in the surviving neurons (Lewy pathology) in different areas of the central and peripheral nervous system (Djaldetti et al., 2009; Beach et al., 2010; Del Tredici and Braak, 2016). Generally PD is considered as a movement disorder, but many patients also suffer from non-motor symptoms (Lee and Koh, 2015), including olfactory and sleep disturbances (Ross et al., 2006; Haehner et al., 2009; Ponsen et al., 2009), depression (Reijnders et al., 2008), cognitive decline with regard to visuospatial perception and working memory (Aarsland et al., 2003), and gastrointestinal dysfunctions (Savica et al., 2009; Jost, 2010; Pfeiffer, 2011; Fasano et al., 2015). These symptoms may precede the motor phase by decades (Abbott et al., 2001; Gao et al., 2011; Chen et al., 2015), have a major effect on the quality of life (Schrag et al., 2000; Martinez-Martin et al., 2011; Müller et al., 2013) and remain undertreated (Chaudhuri and Schapira, 2009).

L-3,4-di-hydroxy-phenylalanine (levodopa) is a precursor of the neurotransmitter dopamine that reduces some of PD motor symptoms since it compensates for dopamine-producing cell loss by enhancing dopamine synthesis in the remaining neurons. The oral supplementation with levodopa is the most efficient drug in the treatment of PD. In the last years, 85% of PD patients were prescribed levodopa (Crispo et al., 2015). However, the drug showed to have several side effects (Schrag and Quinn, 2000). Levodopa does not prevent dopaminergic cell loss, it does not improve all motor symptoms (Sethi, 2008; Anderson et al., 2014) and most of the non-motor symptoms are unresponsive to it (Lee and Koh, 2015). In addition, PD-associated gastrointestinal dysfunction might exacerbate levodopa response fluctuations (Poewe et al., 2010) and on-off oscillations (Hardoff et al., 2001; Müller et al., 2006; Doi et al., 2012; Marrinan et al., 2014).

We recently demonstrated beneficial effects of specific dietary interventions in a mouse model of PD when given therapeutically, i.e., after the full induction of motor symptoms (Perez-Pardo et al., 2017). We showed that rotenone-induced motor, cognitive and gastrointestinal dysfunctions were significantly alleviated by the therapeutic dietary intervention containing both precursors and cofactors for phospholipid synthesis as well as prebiotic fibers (Perez-Pardo et al., 2017). The purpose of the present study is to see whether levodopa is effective in the rotenone model applied and to examine whether there are interactions and/or additive effects between the diet and levodopa.

## Materials and Methods

### Mice

Forty-eight 7-week-old C57BL/6J mice (Charles River, Netherlands) were housed at room temperature under a 12 h light/dark cycle. Food and water were provided *ad libitum*. All animal procedures were approved by the Ethical Committee of Animal Research of Utrecht University, Netherlands (DEC number 2014.I.12.106).

### Surgery

Mice underwent stereotaxic surgery under isoflurane anesthesia as previously described (Perez-Pardo et al., 2018). In brief, a hole was drilled in the skull and a cannula inserted in the right striatum at the following stereotaxic coordinates: AP +0.4, ML −2.0 from Bregma, and DV −3.3 below dura. Mice were injected with 5.4 μg of freshly prepared rotenone solution (dissolved in 2 μl DMSO) or with the vehicle (2 μl DMSO). Ninety-three days after surgery the mice were euthanized by decapitation.

### Levodopa Effectiveness in the Rotenone Mouse Model

In order to investigate whether levodopa treatment could effectively improve motor function in the intrastriatal rotenone mouse model of PD, all animals were tested on the rotarod and in the grip strength test (as described below) 1 h after oral administration of levodopa (20 mg/kg) or vehicle. Thirty minutes before the oral levodopa administration, all animals received a subcutaneous injection of the decarboxylase inhibitor benserazide (6.25 mg/kg). The rotarod tests and the grip strength test were performed on days 26 and 27 after surgery, i.e., when rotenone-induced motor dysfunctions were fully developed (Perez-Pardo et al., 2017) and before the dietary intervention was started. The order of treatments (levodopa and saline) was balanced according to a Latin square design.

### Diets

Mice were fed either the Control diet (CD) or the Active diet (AD), starting 28 days after surgery, i.e., when motor symptoms plateaued, and dietary treatment was continued till the end of the experiment. Animals were divided into four groups of 12 animals (Sham+CD, Sham+AD, Rotenone+CD and Rotenone+AD). Iso-caloric diets were produced by Research Diet Services (Wijk bij Duurstede, Netherlands) and were based on the CD, i.e., the standard animal food for laboratory rodents AIN-93M (Reeves et al., 1993) with 5% fat. For the AD as previously described (Perez-Pardo et al., 2017), uridine (0.51 g/100 g diet) was added and part of the lipid blend of CD was replaced by fish oil, providing DHA (0.75 g/100 g diet) and EPA (0.50 g/100 g diet). The AD also contained supplementary amounts of choline, phospholipids, selenium, folic acid and vitamins B6, B12, C, D and E, above standard levels in the CD. In addition, the cellulose fibers from the CD were replaced by prebiotic fibers (1.5 g/100 g diet GOS, 0.17 g/100 g diet lcFOS, 1.67 g/100 g diet scFOS, and 1.67 g/100 g diet nutriose) in the AD. Most of the above mentioned nutrients are part of the nutritional combination known as Fortasyn Connect (van Wijk et al., 2014).

### Experimental Design

Starting after surgery, rotarod performance was measured every 7 days to analyze the effects of rotenone injection and the dietary intervention over time. These measurements were conducted at least 1 day before animals received levodopa (as described below) to solely analyze the effectiveness of the dietary intervention. From day 65 after surgery onward, i.e., when the AD was shown to have beneficial effects on motor performance in a previous study (Perez-Pardo et al., 2017), until day 93 (4 weeks in total), animals from the four different groups underwent motor function and spatial recognition testing once a week after oral administration of saline or one of three doses of levodopa (5, 10 and 20 mg/kg), the order of which was balanced according to a Latin square design (Table 1). All animals received a subcutaneous injection of the decarboxylase inhibitor benserazide (6.25 mg/kg) 30 min prior to the oral administration of levodopa. The rotarod test, which started 1.5 h after benserazide administration, was followed by spatial recognition test and grip strength test and performed as described below.

**Table 1:** Specification of the order of treatments received by the four different experimental groups according to a Latin square design.

Allocate for each experimental group	WEEK 1	WEEK 2	WEEK 3	WEEK 4
*n* = 3	saline	levodopa 20	levodopa 10	levodopa 5
*n* = 3	levodopa 5	saline	levodopa 20	levodopa 10
*n* = 3	levodopa 10	levodopa 5	saline	levodopa 20
*n* = 3	levodopa 20	levodopa 10	levodopa 5	saline

*This four experimental groups are: Sham+ control food (CD), Sham+ active food (AD), Rotenone+ control food (CD), Rotenone+ active food (AD)*.

### Assessment of Motor Function and Grip Strength

The motor performance of each mouse was assessed in the rotarod test as previously described (Inden et al., 2007). Briefly, mice were placed on an accelerating rod with speeds starting with 2 rpm and gradually increasing to 20 rpm. Time to first fall was recorded with a maximum duration of 300 s. The test was performed in order to assess motor dysfunction development in time, functional recovery during dietary intervention, and the effects of oral levodopa treatment. Muscular forelimb strength was measured using a grip strength tester as described before (Leiter et al., 2011).

### Assessment of Spatial Recognition

Animals’ ability to react to a spatial novelty after a 3-min delay was measured as described before (De Leonibus et al., 2007; Perez-Pardo et al., 2017). In brief, individual mice were submitted to seven consecutive sessions of 6 min with 3-min intersession intervals. Mice were placed into the empty open field during session 1. During sessions 2–4, mice were placed in the open field containing five objects to habituate to the object configuration (habituation phase). During session 5, the object configuration was changed by moving two objects (displaced objects, DO) and leaving the other three objects in the same position (non-displaced objects, NDO). From sessions 2–5, object exploration was evaluated on the basis of the mean time spent by the animal in contact with the different objects. The animals’ ability to selectively react to the spatial change was analyzed by calculating the spatial re-exploration index (DO[S5] − DO[S4] = DO and NDO[S5] − NDO[S4] = NDO). The time the animals interacted with the DO minus the time they interact with the NDO was used for analysis (DO−NDO). In all sessions, the total activity of the animal in the open field arena was measured.

### Intestinal Transit and Colon Length

Intestinal transit function was assessed in all animals as previously described (Perez-Pardo et al., 2017). Thirty minutes before sacrificing the mice, a 2.5% Evans blue solution in 1.5% methylcellulose (0.3 mL per animal) was intragastrically administered. Intestinal transit was measured as the distance from the pylorus to the most distal point of migration of the Evans blue dye after sacrificing the mice. As an indicator of gross inflammation, the length of the colon was measured.

### Tissue Preparation, Immunohistochemistry and Image Analysis

As previously reported (Perez-Pardo et al., 2017), coronal brain slices of 40 μm were sectioned using a cryostat (CM3050, Leica Microsystems). After incubation with 0.3% H_2_O_2_ for 30 min and following blocking serum, sections were incubated overnight with rabbit anti-tyrosine hydroxylase (TH; Santa-Cruz Biotechnology, 1:1,000). Next day, sections were incubated with a biotinylated secondary antibody (Jackson ImmunoResearch, 1:200) for 2 h. The avidin-biotin method was used to amplify the signal (ABC Kit, Vector) and staining was visualized using 0.05% DAB solution. Digital images of immunostained sections were captured with an Olympus BX50 microscope equipped with a Leica DFC 320 digital camera. TH-immunopositive neurons were quantified stereologically on regular spaced sections. Analyses were performed by researchers that were blind to the treatment condition of the sample.

### Statistical Analysis

Results are expressed as mean ± SEM. ANOVA was used to statistically analyze differences between groups, analyzing the effects of the between subject factors surgery (rotenone vs. vehicle) and/or diet (CD vs. AD), and when appropriate the within subject factor treatment (levodopa dose) or time (for the rotarod test performance over time). Results were considered statistically significant when *p* < 0.05. ANOVAs were followed *post hoc* comparison when appropriate. Analyses were performed using SPSS 22.0.

## Results

### Oral Administration of Levodopa Reduced Rotenone-Induced Motor Dysfunction

**Figure 1:**
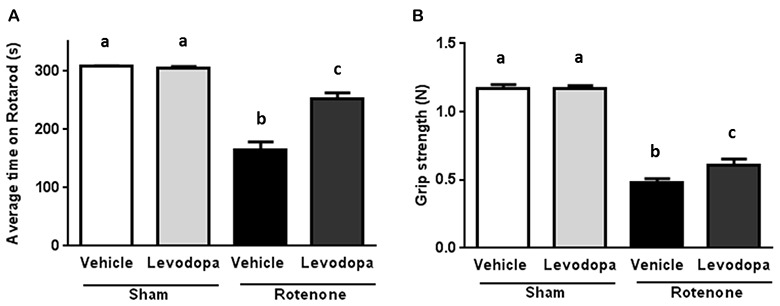
Effects of oral L-3,4-di-hydroxy-phenylalanine (levodopa) treatment (20 mg/kg) on **(A)** rotarod performance and **(B)** grip strength test in sham and rotenone treated groups of mice. Intrastriatal rotenone injection induced motor dysfunction and grip strength loss. Oral Levodopa treatment had beneficial effects on motor function and grip strength in rotenone treated animals. Data are shown as mean ± SEM. Different letters indicate mean values were significantly different (*p* < 0.05).

In order to investigate whether levodopa treatment was effective in the intrastriatal rotenone mouse model of PD and could thus improve motor function, we measured performance on the rotarod (time on rotarod) and the forelimb grip strength 60 min after administration of 20 mg/kg oral levodopa on days 26 and 27 after surgery. Rotenone injection in the striatum negatively affected animals’ ability to remain on the rod (*F*_(1,92)_ = 130.5, *p* < 0.0001) and their forelimb grip strength (*F*_(1,92)_ = 383.4, *p* < 0.0001), as compared to sham-treated mice. There was an overall effect of levodopa on the animals’ motor performances (*F*_(1,92)_ = 24.77, *p* < 0.0001 for the rotarod test and *F*_(1,92)_ = 4.6, *p* < 0.05 for the grip strength test) and an interaction effect between surgery and levodopa treatment in both the rotarod test (*F*_(1,92)_ = 27.66, *p* < 0.0001) and the grip strength test (*F*_(1,92)_ = 4.12, *p* < 0.05). These main and interaction effects indicate an increase in motor function for rotenone-injected mice treated with levodopa (20 mg/kg) as compared to rotenone-injected mice treated with saline, while levodopa administration had no effect on motor performance in sham-treated mice (Figure 1).

### The Dietary Intervention Has a Restorative Effect on Rotenone-Induced Motor Dysfunction

**Figure 2:**
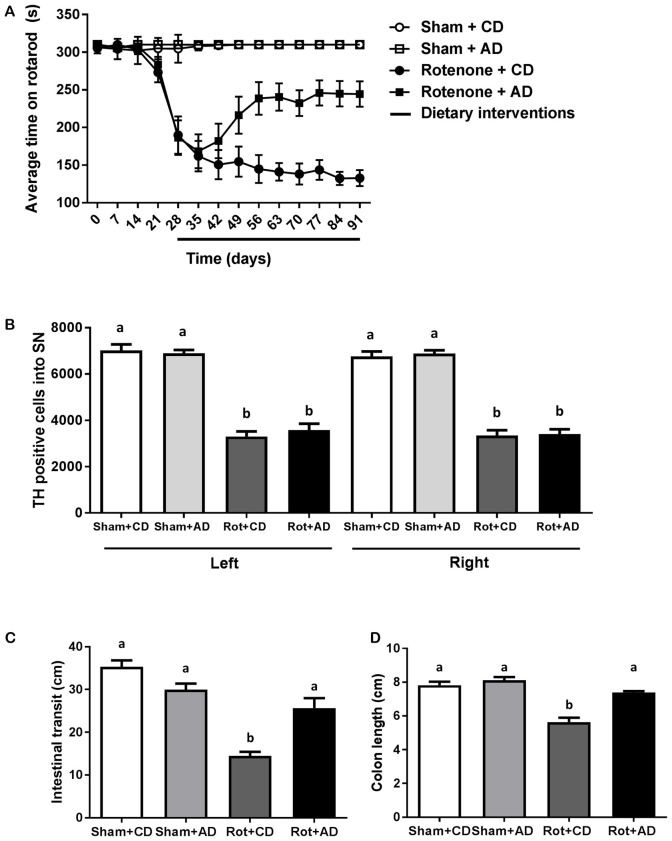
Effects of dietary intervention started after development of full motor dysfunction on: **(A)** rotarod performance over the course of the experiment. Intrastriatal rotenone injection induced a clear motor dysfunction. Dietary intervention started at day 28 after surgery, i.e., after the full development of rotenone-induced motor dysfunction. As compared to Control diet (CD), the Active diet (AD) showed beneficial effects on motor function from day 56 onward, until the end of the experiment. **(B)** The number of dopaminergic cells indicated by the number of tyrosine hydroxylase (TH) immunoreactive cells in the substantia nigra. The dietary intervention had no effect on the number of TH positive cells. **(C)** Intestinal transit indicated by the total distance traveled by the Evans blue dye in the GI tract 30 min after its administration by oral gavage. The AD improved rotenone-induced delayed intestinal transit. **(D)** Colon length. Rotenone injection reduced intestinal transit and colon length. The AD improved rotenone-induced shortening of the colon. Data are shown as mean ± SEM. Different letters indicate mean values were significantly different (*p* < 0.05).

Starting after surgery, rotarod performance was measured every 7 days to analyze the effects of rotenone injection and the dietary intervention over time. There was an overall effect of surgery (*F*_(1,44)_ = 132.08, *p* < 0.0001) and of diet (*F*_(1,44)_ = 6.97, *p* < 0.05) on rotarod performance, i.e., the time spent on the rotarod. Repeated measures showed an effect of time (*F*_(10,440)_ = 36.71, *p* < 0.0001). Rotenone-treated mice developed motor dysfunction over time compared to sham-operated mice (interaction effect surgery × time *F*_(10,440)_ = 39.55, *p* < 0.0001). Moreover, an interaction effect between diet and time (*F*_(10,440)_ = 4.32, *p* < 0.0001), between diet and surgery (*F*_(1,44)_ = 5.48, *p* < 0.05) and between diet, surgery and time (*F*_(10,440)_ = 5.08, *p* < 0.0001) were found. Subsequent analyses per day indicated that rotenone-treated mice showed a decrease in rotarod performance starting from day 28 after surgery onwards compared to sham (*F*_(1,44)_ = 48.63, *p* < 0.0001). In addition, on day 56 after surgery there was a significant effect of the diet (*F*_(1,44)_ = 10.71, *p* < 0.01) on rotarod performance that remained for the duration of the experiment (*F*_(1,44)_ = 31.47, *p* < 0.0001, day 91; Figure 2).

### The Dietary Intervention Has No Effects on Rotenone-Induced Dopaminergic Cell Loss

To investigate the rotenone-induced neurodegeneration, we performed unbiased stereology to estimate the number of TH positive cells in the SN at the end of the experiment (day 93). A significant decrease in the number of TH positive cells in rotenone-treated mice compared to sham-injected mice was observed (*F*_(1,24)_ = 318, *p* < 0.001). There was no main effect of diet and no significant interaction between surgery and diet (Figure 2).

### The Dietary Intervention Has a Restorative Effect on Rotenone-Induced Delayed Intestinal Transit and Reduced Colon Length

As previously demonstrated (Perez-Pardo et al., 2017), the intrastriatal rotenone injection negatively affected intestinal transit as compared to sham-injected mice (*F*_(1,40)_ = 58.94, *p* < 0.0001). There was an overall effect of diet on intestinal transit (*F*_(1,40)_ = 4.24, *p* < 0.05) and an interaction effect between surgery and diet (*F*_(1,40)_ = 25.23, *p* < 0.0001). These main and interaction effects indicate an improved intestinal transit in rotenone-injected mice on AD compared to rotenone-injected mice on CD. In addition, the length of the colon of all the animals was measured as a gross indicator of inflammation. There was a main effect of surgery (*F*_(1,44)_ = 54.20, *p* < 0.0001) and of diet (*F*_(1,44)_ = 26.78, *p* < 0.001), as well as an interaction effect between surgery and diet (*F*_(1,44)_ = 13.40, *p* < 0.001), indicating a smaller decrease in colon length in rotenone-injected mice on the AD compared to the CD (Figure 2).

### The Dietary Intervention Combined With Oral Levodopa Showed Additive Effects on the Disturbed Motor Function of Rotenone-Treated Mice

Between day 65 and day 93, dose-response relationships for levodopa on behavioral performances were assessed in rotenone- and sham-treated animals.

Rotenone treated animals exhibited a decreased rotarod performance compared to sham-treated animals (*F*_(1,176)_ = 425.95, *p* < 0.0001). Rotarod data showed that there was an effect of the diet (*F*_(1,176)_ = 48.28, *p* < 0.0001) and of levodopa treatment (*F*_(3,176)_ = 10.40, *p* < 0.0001). Furthermore, there were interaction effects between surgery and diet (*F*_(1,176)_ = 44.89, *p* < 0.0001), and between surgery and levodopa treatment (*F*_(3,176)_ = 8.94, *p* < 0.0001), indicating that levodopa improved rotarod performance in both rotenone-treated diet groups.

*Post hoc* analyses per group showed that rotenone-treated animals on CD performed significantly better on the rotarod when treated with the highest dose of levodopa (20 mg/kg) compared to animals treated with the lowest dose (5 mg/kg) or saline (*p* < 0.01). Within the rotenone-injected mice on AD, animals treated with levodopa 10 or 20 mg/kg had a better ability to remain on the rod than animals on saline or 5 mg/kg (*p* < 0.001), showing an additive beneficial effect of levodopa on top of the diet.

**Figure 3:**
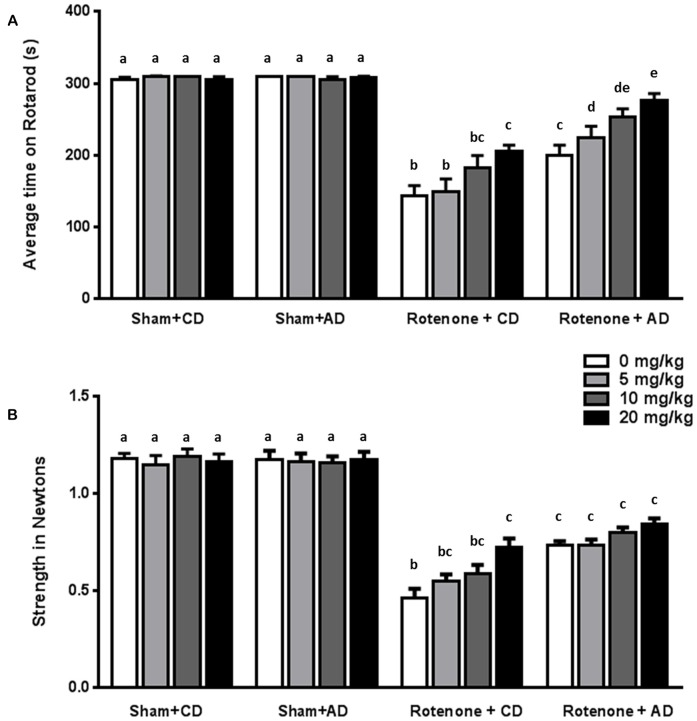
Effects of the dietary intervention and different levodopa doses on **(A)** rotarod performance and **(B)** forelimb grip strength. Both levodopa and dietary treatments alleviated rotenone-induced motor dysfunction. The combined administration of the diet and levodopa showed additive beneficial effects on rotarod performance. Data are shown as mean ± SEM. Different letters indicate mean values were significantly different (*p* < 0.05).

Rotenone treated mice on AD performed significantly better than the animals on CD diet at all doses (0, 5, 10 and 20 mg/kg) (all *p* < 0.05; Figure 3A).

Rotenone-treated mice showed a decrease in grip strength when compared to sham-treated mice (*F*_(1,176)_ = 697.81, *p* < 0.0001). Grip strength was affected by diet (*F*_(1,176)_ = 26.04, *p* < 0.0001) and by levodopa treatment (*F*_(3,176)_ = 3.68, *p* < 0.05). Moreover, there were significant interactions between surgery and diet (*F*_(1,176)_ = 28.38, *p* < 0.0001), and between surgery and levodopa treatment (*F*_(3,176)_ = 5.51, *p* < 0.001), indicating that levodopa improved grip strength in both rotenone-treated diet groups.

More specifically, *post hoc* analyses indicated that rotenone-treated animals on CD showed a significant increase in grip strength when treated with the highest dose of levodopa (20 mg/kg) compared to animals treated with saline (0 mg/kg; *p* < 0.001). No differences were found on grip strength between animals on AD and treated with different doses of levodopa or saline. Rotenone-treated animals on AD performed significantly better than rotenone- treated animals on CD at all levodopa doses (0, 5, 10 and 20 mg/kg; *p* < 0.05; Figure 3B).

### The Dietary Intervention Is Effective in Reducing Rotenone-Induced Spatial Memory Impairments Whereas Levodopa Has No Effects

**Figure 4:**
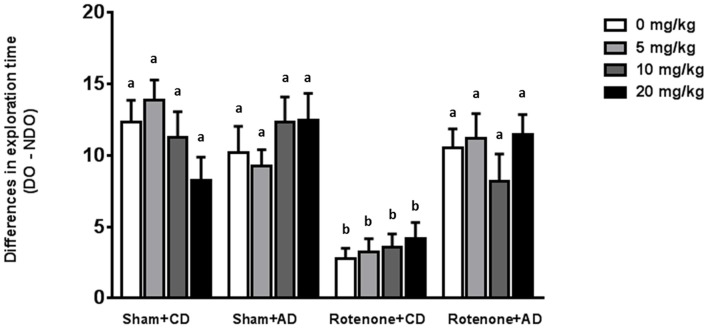
Effects of the dietary intervention and different levodopa doses on spatial object recognition test. Sham-operated animals selectively re-explored the displace object (DO) as compared to the non-displaced object (NDO). Rotenone decreased animals’ ability to react to a spatial novelty. Rotenone-injected animals on the AD showed better spatial discrimination abilities compared to rotenone-injected animals on CD. Levodopa treatments did not affect spatial memory. Different letters indicate mean values were significantly different (*p* < 0.05).

Sham-operated animals selectively re-explored the displace object (DO) as compared to the non-displaced object (NDO), demonstrating that they were able to selectively react to the spatial change. As previously reported (Perez-Pardo et al., 2017), rotenone injection negatively affected the mice ability to react to a spatial novelty (*F*_(1,175)_ = 33.44, *p* < 0.0001). Moreover, there was an effect of the diet (*F*_(1,175)_ = 19.68, *p* < 0.0001) and an interaction effect between surgery and diet (*F*_(1,175)_ = 24.40, *p* < 0.0001). No effects of levodopa treatment on spatial memory were found (*F*_(3,175)_ = 0.091, *p* = 0.96). These main and interaction effects indicated that rotenone-treated mice on AD had a better spatial recognition than rotenone-treated mice on CD independently of the levodopa dose received (Figure 4). No significant differences were found in the total activity in the arena between the different experimental groups.

## Discussion

In the present study, we demonstrate that the oral administration of levodopa positively affects motor symptoms in the intrastriatal rotenone model of PD since the rotenone-injected animals orally treated with levodopa showed a better performance on the rotarod test and an improved forelimb grip strength. Oral levodopa administration did not show any beneficial effect on spatial memory, which is in line with observations in human PD patients where levodopa mainly affects motor symptoms. Together, these observations strengthen the face and construct validity of our model. Furthermore, the present study also demonstrates that a specific dietary intervention (AD) previously shown to normalize rotenone-induced disturbances in motor, gastrointestinal, and spatial memory functioning (Perez-Pardo et al., 2017) has no negative effects on levodopa’s effectiveness on motor function. Moreover, the AD combined with oral levodopa treatment is more effective in reducing motor dysfunction (rotarod performance and grip strength) than the diet or levodopa administered separately. The combined dietary intake of fish oil-derived omega-3 fatty acids, choline, uridine, selenium, folic acid and other nutrients, can increase brain phospholipid levels, synaptic protein levels, neurite outgrowth, dendritic spine formation and dopaminergic neurotransmission (Wang et al., 2005; Sakamoto et al., 2007; Cansev et al., 2008, 2015, 2017). Therefore, it is likely that the AD in the present study enhanced synaptic functioning and neurotransmission in the dopaminergic terminals leading to an improvement of motor performance and an additional effect to that of oral levodopa intake on motor-functioning.

The AD also had positive effects on nonmotor-symptoms that were not affected by levodopa, such as spatial memory functioning, indicating a possible added effect to current treatment. The AD was also shown to have beneficial effects on gastrointestinal functioning in the rotenone model of PD as previously demonstrated (Perez-Pardo et al., 2017). This can be explained by possible enhancing synapse function of enteric neurons but can also be attributed to the beneficial effects of the prebiotic fibers. Besides the induction of growth beneficial bacteria, the prebiotic fibers have been shown to improve immune function and bowel motility (Meksawan et al., 2016). Furthermore, these beneficial effects of the AD in the gastrointestinal tract could be hypothesized to improve levodopa uptake and bioavailability with long-term treatment. Since most of PD patients take levodopa orally, a good functioning of the intestinal tract is needed in order to absorb the drug at a beneficial rate. Several clinical studies revealed that single or multiple doses of levodopa induced delayed gastric emptying in healthy individuals (Robertson et al., 1990, 1992; Waller et al., 1991; Epprecht et al., 2015) and that it might therefore exacerbate the gastrointestinal dysfunction already present in PD patients (Bestetti et al., 2017). Levodopa is absorbed through the duodenum and proximal jejunum by a large neutral amino acid transporter system. Chronic use of oral levodopa is associated with response fluctuations partially due to slow rate of gastric emptying. Delayed gastric emptying in PD patients delays the absorption of levodopa, leading to a lower blood plasma concentrations of the drug and the occurrence of on-off oscillations (Hardoff et al., 2001; Doi et al., 2012; Marrinan et al., 2014). These motor fluctuations, together with dyskinesias are considered as the major side effects of long-term treatment with levodopa (Jankovic, 2005; Müller and Russ, 2006; Poewe et al., 2010). Strategies allowing to reduce the dose of levodopa could potentially reduce the risk of developing such side effects.

In this study, we also replicated previous findings by showing the therapeutic effects of the AD containing phospholipid precursors uridine and DHA in combination with additional nutrients that increase membrane phospholipid synthesis (van Wijk et al., 2014) and prebiotic fibers, in a mouse model of PD given only after full induction of motor symptoms (Perez-Pardo et al., 2017). Our results are complementary to previous observations in other models of neurodegeneration (Jansen et al., 2013; Wiesmann et al., 2016) or acute neurotrauma (Pallier et al., 2015; Wiesmann et al., 2017) where the dietary intervention prevented or rescued both the neuronal connectivity disturbances and the behavioral output deficits.

In summary, the current AD was shown to diminish a broad range of PD-like symptoms in a rotenone mouse model. The AD reduced gastrointestinal dysfunction and did not negatively influence the biological effect of levodopa treatment. In fact, the AD had an additive effect to levodopa on motor performance, next to having beneficial effects on spatial memory, a symptom known to be unaffected by levodopa. Our results suggest that this dietary intervention might confer additional clinical benefits on patients receiving levodopa treatment. Due to the described additive effects, the combination of the diet with the drug might allow a reduction in the doses given to patients, reducing the negative side effects and contributing to a longer beneficial and safe use of the drug.

Future studies are warranted in order to provide further insights on the mechanisms by which the AD has additive effects to levodopa and to better understand how the diet beneficially affect motor and non-motor symptoms.

## Author Contributions

PP-P, LB, TK, NW, AA, JG and AK conceived and designed the experiment. PP-P performed the animal experiments with TK’s assistance. Statistical analysis were designed by PP-P and LB and performed by PP-P. PP-P, LB, TK, NW and AK interpreted the statistical outcome. PP-P wrote the first draft of the manuscript, LB, TK, NW, AA, JG and AK reviewed and critiqued the manuscript. PP-P performed the figures layout. All authors were responsible for the decision to submit the manuscript for publication.

## Conflict of Interest Statement

LB, NW, AA and JG are employees of Nutricia Research, Utrecht, Netherlands. The remaining authors declare that the research was conducted in the absence of any commercial or financial relationships that could be construed as a potential conflict of interest.
